# Intrahost Genetic Diversity of Dengue Virus in Human Hosts and Mosquito Vectors under Natural Conditions Which Impact Replicative Fitness *In Vitro*

**DOI:** 10.3390/v15040982

**Published:** 2023-04-17

**Authors:** Patcharaporn Nonyong, Tipaya Ekalaksananan, Supranee Phanthanawiboon, Hans J. Overgaard, Neal Alexander, Kesorn Thaewnongiew, Vorthon Sawaswong, Pattaraporn Nimsamer, Sunchai Payungporn, Juthamas Phadungsombat, Emi E. Nakayama, Tatsuo Shioda, Chamsai Pientong

**Affiliations:** 1 Department of Microbiology, Faculty of Medicine, Khon Kaen University, Khon Kaen 40002, Thailand; npatcharaporn@kkumail.com (P.N.); tipeka@kku.ac.th (T.E.); supraph@kku.ac.th (S.P.); 2 HPV & EBV and Carcinogenesis Research Group, Khon Kaen University, Khon Kaen 40002, Thailand; 3 Faculty of Science and Technology, Norwegian University of Life Sciences, P.O. Box 5003, 1432 Ås, Norway; hans.overgaard@nmbu.no; 4 MRC International Statistics and Epidemiology Group, London School of Hygiene and Tropical Medicine, London WC1E 7HT, UK; neal.alexander@lshtm.ac.uk; 5 Department of Disease Control, Office of Disease Prevention and Control, Region 7 Khon Kaen, Ministry of Public Health, Khon Kaen 40000, Thailand; kesthaew@hotmail.com; 6 Program in Bioinformatics and Computational Biology, Graduate School, Chulalongkorn University, Bangkok 10330, Thailand; vorthon007.giftedcru@gmail.com; 7 Center of Excellence in Systems Biology, Faculty of Medicine, Chulalongkorn University, Bangkok 10330, Thailand; k.knim@hotmail.com (P.N.); sp.medbiochemcu@gmail.com (S.P.); 8 Department of Biochemistry, Faculty of Medicine, Chulalongkorn University, Bangkok 10330, Thailand; 9 Mahidol-Osaka Center for Infectious Diseases (MOCID), Faculty of Tropical Medicine, Mahidol University, Bangkok 10400, Thailand; juthamas@biken.osaka-u.ac.jp (J.P.); emien@biken.osaka-u.ac.jp (E.E.N.); 10 Research Institute for Microbial Diseases, Osaka University, Osaka 565-0871, Japan

**Keywords:** dengue virus, *Aedes aegypti*, intrahost genetic diversity, selection pressure, host switching, replication kinetics

## Abstract

Dengue virus (DENV) is an arbovirus whose transmission cycle involves disparate hosts: humans and mosquitoes. The error-prone nature of viral RNA replication drives the high mutation rates, and the consequently high genetic diversity affects viral fitness over this transmission cycle. A few studies have been performed to investigate the intrahost genetic diversity between hosts, although their mosquito infections were performed artificially in the laboratory setting. Here, we performed whole-genome deep sequencing of DENV-1 (*n* = 11) and DENV-4 (*n* = 13) derived from clinical samples and field-caught mosquitoes from the houses of naturally infected patients, in order to analyze the intrahost genetic diversity of DENV between host types. Prominent differences in DENV intrahost diversity were observed in the viral population structure between DENV-1 and DENV-4, which appear to be associated with differing selection pressures. Interestingly, three single amino acid substitutions in the NS2A (K81R), NS3 (K107R), and NS5 (I563V) proteins in DENV-4 appear to be specifically acquired during infection in *Ae. aegypti* mosquitoes. Our *in vitro* study shows that the NS2A (K81R) mutant replicates similarly to the wild-type infectious clone-derived virus, while the NS3 (K107R), and NS5 (I563V) mutants have prolonged replication kinetics in the early phase in both Vero and C6/36 cells. These findings suggest that DENV is subjected to selection pressure in both mosquito and human hosts. The NS3 and NS5 genes may be specific targets of diversifying selection that play essential roles in early processing, RNA replication, and infectious particle production, and they are potentially adaptive at the population level during host switching.

## 1. Introduction

Dengue is a mosquito-borne viral infectious disease that is currently transmitted in more than 125 countries worldwide, with an estimated 100 million symptomatic infections and 10,000 deaths per year [1,2]. Transmission between humans is via *Aedes* mosquitoes, which thrive in tropical and subtropical regions [2,3]. Persistent public health problems due to the disease include annual re-emergence with increased disease severity. Effective vaccines against all four serotypes are unavailable, and there is an unmet need for specific antiviral therapeutic drugs [4,5]. DENV is a single-stranded, positive-sense RNA virus of the genus *Flavivirus*, family *Flaviviridae*. The DENV genome is approximately 11 kb in length, and encodes a single polyprotein that is cleaved by cellular and viral proteases into three structural proteins (capsid, pre-membrane, and envelope) and seven non-structural proteins (NS1, NS2A, NS2B, NS3, NS4A, NS4B, and NS5) [6,7]. The non-structural proteins are responsible for viral replication, reshaping the inner cell organization, polyprotein maturation, and facilitating the virus’ evasion of the host immune system [8,9].

The serotypes DENV-1 to -4 are transmitted to humans by two *Aedes* species [3]. *Aedes (Ae.) aegypti* is the primary vector and *Ae. albopictus* is a secondary vector [10]. Once the virus enters the mosquito vector in a viremic blood meal, it replicates in the midgut, is released into the hemocoel, and disseminates to other tissue via hemolymph, finally reaching the salivary gland to transmit to a new host in a subsequent bite [11]. There are two main antiviral response strategies against DENV in the mosquito: antimicrobial peptides (AMP) and RNA interference (RNAi) [11].

The lack of proofreading activity of DENV RNA-dependent RNA polymerase results in high error rates in RNA synthesis during viral replication, which promotes the accumulation of DENV genetic heterogeneity in individual hosts and population complexity, sometimes termed quasispecies [6]. High intrahost genetic diversity is believed to help RNA viruses to replicate under strong selective pressure from hosts, which may impact viral pathogenesis, transmission, tropism, and host control [10,12,13]. Genetic variability may also affect the outcomes of disease treatment. For example, the rapid emergence of drug-resistant strains in human immunodeficiency virus (HIV) and hepatitis C virus (HCV) requires combination therapies for preventing disease [14,15,16].

The life cycle of mosquito-borne viruses, such as DENV, within alternating vertebrate and invertebrate hosts has been shown to cause significant differences in variant population frequency among hosts [12]. Although the virus replicates within a single host, intrahost variants have been driven during replication by several evolutionary mechanisms, such as positive or negative selection, genetic drift, and bottlenecks [17]. Recently, DENV genetic diversity has been assessed in laboratory settings by tracking genetic variants transmitted from humans to mosquitoes, either by intrathoracically inoculating the virus isolated from the viremic blood of patients to mosquitoes [12], or by feeding the mosquitoes with viremic blood [17]. The authors found that most of the mutations occurring during human infection revert back to the original state once transmitted to the mosquito [12,17]. However, studies on selection pressure on the DENV genome in humans and in mosquitoes show conflicting results. One study found that purifying selection of the DENV-1 genome occurred in both humans and mosquitoes [12], whereas other studies have shown that diversifying selection occurred in humans and purifying selection in mosquitoes [17]. It is unclear what exactly happens to the intrahost diversity of DENV during replication in humans and mosquitoes in natural infection.

To address this issue, we performed high-throughput sequencing of DENV whole genomes extracted directly from dengue patients and naturally infected mosquitoes to characterize DENV intrahost genetic diversity. Furthermore, the biological relevance of the *Ae. aegypti*-specific substitutions was investigated *in vitro*.

## 2. Materials and Methods

### 2.1. Ethics Statement

The study protocols were approved by the Khon Kaen University Ethics Committee for Human Research (KKUEC, no. HE611454). Informed consent was obtained in writing from all participants before sample collection. In the case of minor or child participants, written informed consent was sought from their parents or legal guardians. All methods were performed in accordance with the World Medical Association Declaration of Helsinki.

### 2.2. Study Population

Human viremic plasma and dengue virus-infected field-caught *Ae. aegypti* mosquito samples used in the present study originated from our previous dengue case–control study in Northeastern Thailand in 2016–2018 [18]. Enrolled patients presented with suspected dengue at local hospitals and plasma samples were collected during the acute phase. Mosquitoes were collected from these patients’ houses immediately after the case report. Of the 103 DENV-positive patients and 53 infected pooled mosquito samples previously described [19], we successfully performed whole-genome sequencing of DENV-1 (plasma *n* = 9, mosquitoes *n* = 2) and DENV-4 (plasma *n* = 11, mosquitoes *n* = 2) samples.

### 2.3. RNA Extraction, Library Preparation, and Sequencing

Next-generation whole-genome sequencing of human- and mosquito-derived DENV-1 and DENV-4 samples was performed as previously described [20]. Viral RNA was directly extracted from human plasma or lysates of mosquito pooled samples using QIAamp viral RNA mini kits (Qiagen, Hilden, Germany), and cDNA was synthesized from extracted viral RNA using the SuperScript III first-strand synthesis system (Invitrogen, Waltham, MA, USA) according to the manufacturer’s instructions. The entire DENV genome was PCR-amplified in two overlapping fragments using PrimeSTAR GXL DNA polymerase (Takara, Shiga, Japan). A set of primer sequences used in the present study is shown in Table S1. Amplicon concentrations were measured and equalized to 0.2 ng/µL using the Qubit fluorometer (Thermo Fisher Scientific, Waltham, MA, USA), and DNA libraries were constructed using the Illumina Nextera XT library preparation kit (Illumina, San Diego, CA, USA). The pair-end sequencing of prepared libraries was performed on the Illumina MiSeq (Illumina, USA) using a MiSeq v2 kit (500 cycles) reagent cartridge. After sequencing, reads were demultiplexed by the instrument, and raw sequence data were generated as FASTQ files. All raw data have been deposited into the NCBI sequence read archive (BioProject accession number PRJNA855615).

### 2.4. Mapping and Single-Nucleotide Variant (SNV) Calling

All FASTQ files of all samples were imported from MiSeq into the CLC Genomics Workbench v10.1.1 (CLC Bio, Aarhus, Denmark). For quality control, a quality score greater than or equal to 30 (≥Q30) and a read length longer than 100 nucleotides were the criteria used to trim reads. The processed reads of each sample were mapped to the reference genome of DENV-1 (NC_001477) and DENV-4 (NC_002640) to build an initial consensus sequence. SNV calling was performed on each sample with a minimum average coverage of 100 reads per position and with a central base quality score of ≥Q30. Only variants presenting at a frequency ≥1% were considered for further analysis. The selection pressure experienced by DENV in each host type was estimated in terms of the ratio of nonsynonymous (dN) to synonymous (dS) substitutions (dN/dS) using the Nei–Gojobori method (Jukes–Cantor model) implemented in MEGA X [21]. The results are shown in Supplementary Figure S1.

### 2.5. Cells, Infectious Clone, and Antibody

C6/36 cells (*Aedes albopictus* cell line) were cultured at 28 °C in L-15 medium (Gibco, Waltham, MA, USA) supplemented with 10% FBS and 10% tryptose phosphate broth (Sigma, St. Louis, MO, USA). Vero cells were grown in Dulbecco’s Modified Eagle Medium (DMEM) (Gibco, Waltham, MA, USA) supplemented with 10% fetal bovine serum (FBS) (HyClone Laboratories, Logan, UT, USA) at 37 °C with 5% CO_2_. A DENV-4 infectious clone (DENV-4 IC, pmMW/rR05-167) was kindly provided by Professor Takeshi Kurosu (Research Institute for Microbial Diseases (RIMD), Osaka University, Japan), produced using a two-plasmid ligation strategy as described previously [22]. The primary antibody used in this study was a goat anti-mouse IgG conjugated with Alexa Fluor 488 (Thermo Fisher Scientific, USA).

### 2.6. Site-Directed Mutagenesis

To examine the biological relevance of the *Ae. aegypti*-specific mutations, we generated mutants including A3721G (K81R), A4843G (K107R), and A9249G (I563V) in the context of the genome-length RNA of DENV-4. Three mutants were obtained by the site-directed mutagenesis of DENV-4 IC through PCR with the In-Fusion^®^ HD Cloning Kit (Clontech, CA, USA) according to the manufacturer’s instructions, using mutagenic primer sets (Table 1). Mutant DENV-4 IC DNA was then amplified in *E. coli* stella and plasmids harvested using Qiagen^®^ Plasmid Midi Kits (Qiagen, Germany). All these point mutations, and the control (wild type) genome were confirmed by Sanger sequencing. Transcription of mutant and control DENV-4 IC plasmids was carried out by linearizing with NotI and *in vitro* transcription using the mMessage mMachine kit (Ambion, Austin, TX, USA). Transcription reactions were incubated at 37 °C for 3 h, followed by the addition of DNase I to remove the DNA template. The resulting RNA was precipitated with lithium chloride, washed with 70% ethanol, re-suspended in RNase-free water, assessed using a Nanodrop instrument (Thermo Fisher Scientific, Waltham, MA, USA), and stored at −80 °C. Wild-type and mutant DENV-4 IC RNA were added onto a 90% confluence seeded Vero:C6/36 (1:1) mixture cell monolayer along with DMRIE-C transfection reagent (Thermo Fisher Scientific, Waltham, MA, USA) according to the manufacturer’s instructions. After incubation at 37 °C for 6 h, the supernatant was discarded and replaced with Dulbecco’s Modified Eagle’s Medium (DMEM) supplemented with 2% FBS and 10% tryptose phosphate broth. Supernatants were collected from day 3 to 7 post-transfection, aliquoted, and stored at −80 °C. DENV-4 infectious particles were quantified by Indirect Immunofluorescence Assays. The virus in the supernatant from cultures of the transfected cells was passaged a second time in C6/36 cells to produce a virus of sufficient titer for subsequent experiments. The recovered recombinant virus stocks were also completely sequenced to confirm that the introduced mutations had not reverted and that other modifications had not occurred.

### 2.7. Indirect Immunofluorescence Assays

To measure the virus titer, Vero cells were seeded in 96-well plates at 2 × 10^4^ cells/well and, on the next day, incubated with serial dilutions of virus samples in DMEM without FBS at 37 °C for 2 h. After incubation, cells were overlaid with MEM containing 1.5% methylcellulose and 2% FBS. Three days after infection, the cells were fixed with 3.7% *v*/*v* formaldehyde/PBS for 1 h, and permeabilized with 0.5% *v*/*v* Triton X-100 (Sigma-Aldrich, St. Louis, MO, USA) in PBS for 10 min. After 1 h of incubation in a blocking buffer containing 1% FBS and 0.05% Tween-20 in PBS, the cells were treated with a mouse monoclonal antibody 4G2 for 1 h and washed three times with PBS. The cells were then incubated with Alexa Fluor 488 goat anti-mouse IgG for 1 h in the blocking buffer. Finally, the cells were washed three times with PBS, and the number of foci was counted under a fluorescence microscope (Olympus, Tokyo, Japan).

### 2.8. Viral RNA Quantification

The infectivity of viral RNA was determined by transfection of 2 µg transcribed RNA onto monolayers of Vero or C6/36 cells in 12-well plates, in triplicate wells, with DMRIE-C transfection reagent (Thermo Fisher Scientific, Waltham, MA, USA) according to the manufacturer’s instructions. The cells were incubated in a medium containing 2% FBS and culture supernatants were collected daily until 10 days post-transfection (d.p.t.). The viral RNA was isolated from 140 µL aliquots of culture supernatant using the QIAamp Viral RNA Mini Kit (Qiagen, Hilden, Germany), according to the manufacturer’s protocols. RNA was reverse-transcribed into cDNA by a specific (D2-R) primer [23] using the SuperScript^®^ III first-strand synthesis system (Invitrogen, Waltham, MA, USA), according to the manufacturer’s instructions. cDNA was quantified using real-time PCR and an SYBR green-based assay using a set of dengue group-specific primers: DN-F (5′-CAA TAT GCT GAA ACG CGA GAG AAA-3′ and DN-R (5′-CCC CAT CTA TTC AGA ATC CCT GCT-3) [24]. The reaction conditions were as follows: 35 cycles of 95 °C for 1 min, 55 °C for 30 s, and 72 °C for 3 min. This was followed by a melting curve analysis using the Applied Biosystems^®^ 7500 Real-Time PCR machine (Applied Biosystems, Foster City, CA, USA). The DENV-4 IC (pmMW/rR05-167) plasmid was used as a standard. Negative controls (reaction without cDNA template) were included with each amplification reaction. The viral copy numbers were analyzed using one-way analysis of variance (ANOVA) and Tukey’s correction for multiple comparisons, with *p*-values less than 0.05 (*p* < 0.05) being considered statistically significant.

### 2.9. Replication Kinetics

Confluent monolayers of Vero or C6/36 cells in 12-well plates were inoculated with either WT or mutant-derived infectious clones at a multiplicity of infection (MOI) of 0.01 in triplicate wells. One hundred microliters of the virus were added to each well of the 12-well plates. After 2 h of adsorption (5% CO_2_ at 37 °C for Vero cells and at 28 °C for C6/36 cells), the cells were washed three times with PBS. The cells were incubated in DMEM medium containing 2% FBS, and culture supernatants were collected daily until day 10 post-infection (d.p.i.). The infectious DENV produced in this way was quantified by immunofluorescence assays as described above.

### 2.10. Data Analysis and Software

All the graphs and statistical analyses were performed using the GraphPad Prism software version 7.0. The proportions of nonsynonymous mutations per nonsynonymous site (dN) and synonymous mutations per synonymous site (dS) of each coding gene were calculated using the MEGA software version 10.0. The statistical comparisons of selection pressure between two hosts were performed using the Mann–Whitney test. Protein structures were visualized and amino acid mutations were mapped on the structure using the PyMOL software. Infectivity and replication kinetics were compared using one-way analysis of variance (ANOVA), with the Tukey procedure. Values of *p* less than 0.05 are considered statistically significant.

## 3. Results

### 3.1. Intrahost Genetic Diversity of DENV-1 and -4 in Humans and Mosquitoes

**Figure 1:**
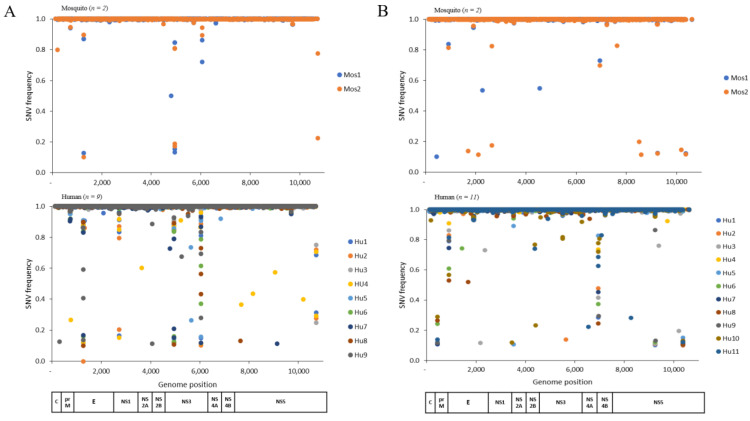
Intrahost genetic diversity of DENV. The variant frequency is plotted on the y-axis for DENV-1 (**A**) and DENV-4 (**B**), with genome position on the x-axis. This analysis was completed for each host type: humans (Hu) and mosquitoes (*Ae. aegypti* Mo). C: capsid, prM: precursor membrane, E: envelope, NS: non-structural protein, SNV: single-nucleotide variant.

The samples that passed the quality control filter criteria described above were analyzed for variant frequency along the viral genome. Variant frequency in the DENV-1 and DENV-4 genomes was analyzed to evaluate the intrahost genetic diversity within individual samples of humans and mosquitoes (Figure 1). The total numbers of SNVs along the DENV-1 genome in human and mosquito samples were 877 and 198, respectively (Figure 1A). For DENV-4, the corresponding numbers were 1096 and 868 (Figure 1B). Moreover, within each viral gene region in each host, the within-sample variability did not seem to have any constant pattern. In DENV-1 human samples, several genes seemed to present higher variability, such as NS2A, NS4A, and NS2B, while, in mosquito samples, it was found in NS2A, prM, NS1, and NS4A. For DENV-4, in human samples, higher variability was found in NS2A, NS4A, NS4B, and NS1, whereas, in the mosquito samples, this was found in NS2A, prM, and NS1.

### 3.2. Specific Nonsynonymous Substitution Is Acquired in Ae. aegypti Mosquito

**Figure 2:**
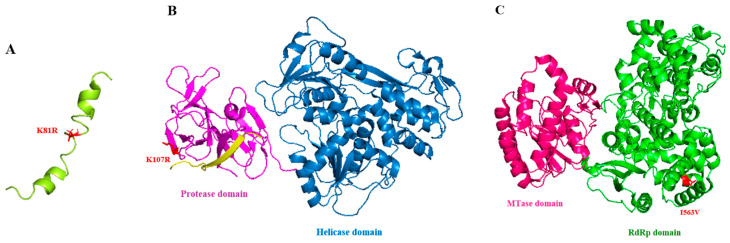
The crystal structure of DENV NS2A, NS3, and NS5 proteins. (**A**) Ribbon representation of the protein structure of NS2A (first transmembrane segment, PDB 2M0S). (**B**) Ribbon representation of complete NS3 (PDB 2VBC) protein structure. A small yellow beta sheet represents the NS2B cofactor, purple represents the NS3 protease domain, and a large blue molecule represents the helicase domain of the NS3 protein. (**C**) Ribbon representation of the complete NS5 (PDB 4V0Q) protein structure: pink represents the MTase domain and green represents the RdRp domain. The locations of substitutions and residues on each protein are denoted in red. Structure figures were prepared using PyMOL.

Since DENV-4 exhibited higher genetic variation than DENV-1, we focused on DENV-4 consensus sequences to identify patterns of mutations that suggest a potential relationship between host type and adaptation. We assessed the nonsynonymous substitutions (NS) commonly arising during mosquito vector transmission (Table 2). Twenty-four substitutions were commonly found in mosquito-derived viruses, with fewer being found in human-derived viruses (Table 2). Among them are two substitutions in DENV-4 consensus sequences that occurred in 100% of mosquito-derived viruses, but were found in only 18.2% and 0% of human-derived viruses. These were the NS2A (A3721G) and NS3 (A4843G) proteins, respectively. Another four substitutions, the C (G186A), E (G1602A), E (C1637T), and NS5 (A9249G) proteins, were found to occur in 100% of mosquito-derived viruses, but only 45% of human-derived viruses. Thus, six substitutions seem to be commonly involved in the mosquito adaptation of DENV-4. Among these, we selected three that occurred in nonstructural proteins, namely the NS2A (A3721G), NS3 (A4843G), and NS5 (A9249G) proteins, to assess the relationship of substitution regarding DENV-4 replicative fitness in further site-directed mutagenesis experiments. Furthermore, we constructed a DENV protein structure to identify the positions of these three substitutions in the protein crystal structure using PyMOL. The consensus change observed at nucleotide position A4843G corresponds to amino acid 81 (K81R), which is located in the first transmembrane segment of the NS2A protein (Figure 2A). The A4843G substitution falls within the protease domain of the NS3 protein, resulting in an amino acid change at position 107 (K107R) (Figure 2B). Another substitution at nucleotide position A9249G, located within the RNA-dependent RNA polymerase (RdRp) domain of the NS5 protein, results in an amino acid change at position 563 (I563V) (Figure 2C). These substitutions on the DENV-4 genome seem to be essential and specific responses to the *Ae. aegypti* mosquito during transmission.

### 3.3. RNA Production from DENV-4 Infectious Clone

**Figure 3:**
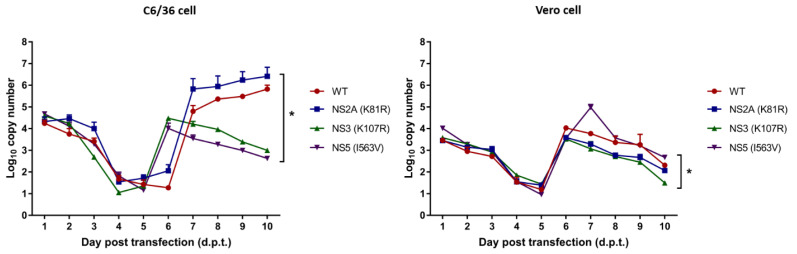
Infectivity of infectious clones containing genome length RNA of wild type (WT), NS2A (K81R), NS3 (K107R), and NS5 (I563V) mutants in C6/36 cells and Vero cells. The viral copy numbers were measured from culture supernatants by RT-qPCR at 1 to 10 d.p.t. Results are expressed as the mean and SD of triplicate experiments. Statistically significant differences between each group are indicated by * *p* < 0.05 (one-way ANOVA with multiple comparisons, Tukey’s post-test).

To examine the infectivity of the infectious clone, we transfected the RNA transcript of the DENV-4 R05-167 wild type and three mutants, NS2A (K81R), NS3 (K107R), and NS5 (I563V), into mosquito C6/36 cells or mammalian Vero cells. The transfected cells were monitored and their RNA synthesis was measured from day 1 to day 10 post-transfection (d.p.t.). DENV RNA in the culture supernatants of WT- and mutant-transfected cells showed different amounts of RNA (Figure 3). In C6/36 cells, NS3 (K107R) and NS5 (I563V) reached the maximum RNA copy number at day 6 with 4.5 and 4.0 log_10_ copies, respectively. WT and NS2A (K81R) peaked later, at day 7, and higher, with 4.8 and 5.8 log_10_ copies, respectively (Figure 3). In Vero cells, the highest amounts of viral RNA were produced in the wild type and three mutants with peaks of 4–5 log_10_ copy number on days 6, before decreasing over days 7–10 (Figure 3). Results demonstrated significantly different viral copy numbers between WT and three mutants at 1–10 d.p.t. (* *p* < 0.05 one-way ANOVA with multiple comparisons, Tukey’s post-test). The Sanger sequencing results of the mutant viruses revealed no change at the nucleotide substitution point that originated from mutated infectious clones, NS2A (A3721G), NS3 (A4843G), and NS5 (A9249G) (Figure S2). Additional qualifications about the replicative fitness of these mutants may be appropriate, while further studies are required for additional replication-defective genome detection as an additional technical control. These results suggest that mutations in the NS3 (K107R) and NS5 (I563V) proteins seem to impact viral infectivity and have an effect on the prolonged replication kinetics in the early phase in both Vero and C6/36 cells.

### 3.4. Replication Kinetics

**Figure 4:**
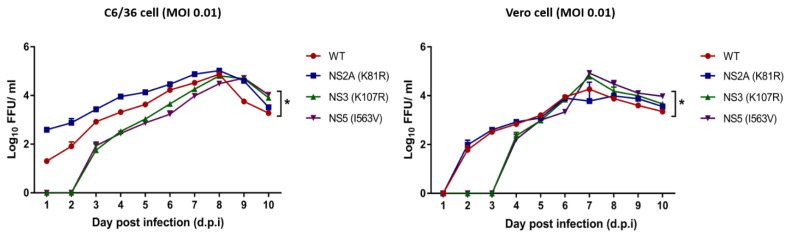
Replication kinetics of DENV-4 wild type (WT), NS2A (K81R), NS3 (K107R), and NS5 (I563V) viruses in C6/36 cells and Vero cells. *In vitro* experiments were conducted at an MOI of 0.01. The viral titer of the cell culture supernatant was determined from 1 to 10 d.p.i. by immunofluorescence focus assays (IFA). Results are expressed as the mean and SD of triplicate experiments. Statistically significant differences (one-way ANOVA test) between each group are indicated by * *p* < 0.05 (one-way ANOVA with multiple comparisons, Tukey’s post-test).

To better understand the underlying mechanism, we examined the replication kinetics of the mutants in mosquito C6/36 cells or mammalian Vero cells. In C6/36 cells, there were no detectable NS3 (K107R) and NS5 (I563V) viruses until 3 d.p.i., with 1.8 and 1.9 log_10_ FFU/mL, respectively, while WT and NS2A (K81R) viral titers increased from 1 d.p.i. and reached 1.3 and 2.5 log_10_ FFU/mL, respectively. The results revealed significantly lower viral titers in NS3 (K107R) and NS5 (I563V) viruses at 1–7 d.p.i. in C6/36 cells (* *p* < 0.05 one-way ANOVA with multiple comparisons, Tukey’s post-test), and then higher peak titers after 8 d.p.i. (Figure 4). For Vero cells, the NS3 (K107R) and NS5 (I563V) viruses were also undetectable at the initial time points. Then, at 4 d.p.i., the viral titers reached 2.3 and 2.2 log_10_ FFU/mL, respectively. Interestingly, the titers of NS3 (K107R) and NS5 (I563V) were significantly higher than those of WT from 7 to 10 d.p.i. in Vero cells (* *p* < 0.05 one-way ANOVA with multiple comparisons, Tukey’s post-test) (Figure 4). These results demonstrated that the NS3 (K107R) and NS5 (I563V) viruses were undetectable over days 1–2 and days 1–3 post-infection in mosquito C6/36 cells and Vero cells, respectively, and they have lower viral titers at early replication when compared to the NS2A (K81R) and WT viruses. Although the NS3 (K107R) and NS5 (I563V) viruses had lower viral titers in the early phase of replication, the viral titer became higher than that of WT in the late phase of replication after 8 and 7 d.p.i. in C6/36 cells and Vero cells, respectively. Therefore, the relatively low yield of the infectious virus of NS3 (K107R) and NS5 (I563V) mutants was accompanied by RNA synthesis (Figure 4) in the early phase of infection but increased in the late phase (Figure 4). The results suggest that mutations in NS3 (K107R) and NS5 (I563V) might be involved in DENV-4 adaptation during mosquito infection.

## 4. Discussion

Intrahost genetic diversity is thought to impact the viral fitness and adaptive potential of RNA viruses [25,26,27,28,29,30], and specifically DENV [12,17,31], during alternate replication between two different hosts. In the present study, we analyzed the intrahost genetic diversity directly from dengue patients and naturally infected field-caught *Ae. aegypti* mosquitoes from the patients’ houses by using next-generation whole-genome sequencing. In addition, we evaluated the influence of specific mutations of mosquito-derived DENV-4 on *in vitro* replication kinetics.

We observed a higher SNV frequency in humans than in mosquitoes for DENV-1 and -4 virus populations (Figure 1). A previous study reported that the vast majority (>90%) of SNVs in DENV-2 populations were lost during virus transmission from humans to mosquitoes [17]. It has been suggested that DENV-1 SNVs accumulating in humans predominantly revert back to the original state when the virus transmits to mosquitoes [12]. Such findings support the idea that most mutations within humans are deleterious for viral fitness within mosquitoes [17,32]. Moreover, the variability of both DENV serotypes within human and mosquito-derived samples showed a lack of consistent patterns.

Twenty-four amino acid substitutions in the DENV-4 consensus genome sequences were found to be primarily changes in naturally infected mosquitoes rather than humans (Table 2). Of these, three amino acid substitutions in the nonstructural proteins NS2A (K81R), NS3 (K107R), and NS5 (I563V) were selected for further *in vitro* experiments in order to measure the viral replication kinetics. A substitution in the NS2A (K81R) protein was found in the pTMS3 trans-ER membrane domain, located upstream of the helix breaker that separates two helical transmembrane segments (Figure 2A). Another substitution in the NS3 (K107R) protein occurred in its N-terminal protease domain (Figure 2B), which cleaves the viral polyprotein precursor into individual nonstructural proteins and the C-terminal RNA helicase domain involved in viral RNA synthesis [33]. NS5, the largest and the most conserved DENV protein, a bifunctional enzyme with an MTase domain and an RdRp domain, is involved in viral genome replication and its capping [34,35]. The RdRp domain is composed of three subdomains, namely the palm, thumb, and finger subdomains, which are conserved across the viral protein structure [36]. The amino acid substitution I563V in NS5 falls within the RdRp domain at the junction of the palm subdomain, which is close to the active site of the enzyme (Figure 2C). Several investigations have found mutations in mosquito-derived variants in the NS3 protein and 3′ UTR region of DENV-2, and in the NS5 protein of DENV-1 [12,17], suggesting that these three genes experience strong immune pressure from the mosquito immune system.

To assess the viral replicative fitness of these three substitutions that were specifically detected in mosquitoes, we engineered individual mutations into a DENV-4 infectious clone and conducted an *in vitro* study in mosquito C6/36 cells and mammalian Vero cells. In the infectious clone infectivity evaluation, the viruses required 6 days of culture post-transfection for DENV RNA levels to increase, and it appears that the values for the first 5 days of culture are likely the result of the detection of input RNA (Figure 3). Considering this limitation, further studies should include an additional replication-defective (‘GND’) genome for comparison in these experiments as an additional technical control to enable a straightforward interpretation of how the replication-competent viral RNA levels compare to the input RNA levels that result from transfection. The replication of NS3 and NS5 mutants was delayed in the eclipse phase (day 1–3 post infection) in Vero and C6/36 cells (Figure 4). However, viral production rates became similar to those of the NS2A mutant and WT in the late phase of replication. It is notable that two mutated residues in the NS3 and NS5 proteins interfered with viral replication in the early phase, prolonging the eclipse phase, while in NS2A, there was no effect on viral growth kinetics. The most prominent finding from these studies is that delayed growth tended to support frequently detected mutations, which is consistent with our data on the strength of diversifying selection pressure. Our results are consistent with a previous mutagenesis study of DENV-2 NS2A within pTMS3 (amino acids 69 to 93), which revealed that the substitution of R84A has no effects on viral RNA synthesis but blocks the intracellular formation of infectious virions [37]. Moreover, other mutagenesis studies of NS2A in other transmembrane segments, pTMS4 to pTMS8, have shown an impairment of virion assembly without specifically affecting viral RNA synthesis [38,39]. It has been found that the disruption of the DENV-2 NS2B-NS3 protease domain decreases dengue virus infection by 80% [40]. In a yellow fever virus study, a mutation in the NS2B-NS3 protease left the virus unable to infect target cells [41]. Consistent with our results, several previous investigations suggest that the disruption of NS2A, NS2B-NS3pro, and NS5 protein function affects viral replication [9,40].

DENV can evade the host’s immune response during replication in two main ways: (i) controlling the host’s innate immune response, such as stress that triggers cell death, and surviving autophagy to facilitate viral replication; (ii) directly inhibiting the innate immune response’s signaling cascade via nonstructural proteins and also inhibiting the signaling pathway of RNAi and IFN-α/β induction [42]. Several lines of evidence suggest that vector-borne flaviviruses are persistent between cycles of either vertebrate or invertebrate (tick or mosquito) hosts [43]. Mutations in the C, E, NS1, NS2A, NS2B, and NS5 proteins of WNV have been associated with viral persistence [44]. Similarly, it has also been reported that mutations in the NS4B, NS2A, and NS3-NS4A proteases affect IFN induction by blocking and/or interfering with mosquito-borne viruses, Kunjin virus, and HCV, respectively [45,46,47,48,49]. Our results demonstrate that mutations in the NS3 (K107R) and NS5 (I563V) proteins result in a prolonged eclipse phase of viral replication, which might be involved in the immune evasion in the mosquito system.

Our study provides useful information on specific substitution regions in viral genomes during host switching, although it has some limitations. We were only successful with whole-genome sequencing in a small number of mosquito samples. Two overlapping amplicons were prepared from the samples without virus isolation to avoid genetic changes due to the culture process. Shorter fragments of PCR amplicons have been suggested to be more suitable for whole-genome sequencing [12,17], which we will consider in future studies. Another limitation is that the DENV from humans and mosquitoes may originate from different places and so they are not necessarily directly related. Although we tried to collect samples at the same time from the patient and from the mosquito at the patient’s residence, the patient may well have been infected elsewhere, e.g., at work, school, or while traveling. However, DENV samples were of the same genotype within each serotype, namely genotype I for DENV-1 and -4 positive samples [19].

In conclusion, differing selection pressure in vertebrate host and invertebrate vectors drives DENV’s diversity and hence replicative fitness. NS3 and NS5 may be specific targets of diversifying selection during replication in mosquito vectors, playing essential roles in early processing, RNA replication, and infectious particle production. These two genes may favor adaptive changes at the population level during host switching, especially in mosquitoes. This could be a target used for the exploration and monitoring of emergent DENV phenotypes.

## Figures and Tables

**Table 1:** Mutagenic primers used for construction of NS2A, NS3, and NS5 mutant plasmids.

Name	nt ^c^	aa ^d^	Primer Sequence (5′→3′)
∆NS2A-F ^a^	A3721G	K81R	CCATCATGGCAGTGTTCAGGATGTCAC
∆NS2A-R ^b^			CGTATCCTGGTGACATCCTGAACACTG
∆NS3-F ^a^	A4843G	K107R	GAACCAGGAAAAAATCCGAGACATGTCC
∆NS3-R ^b^			GTTTCGTTTGGACATGTCTCGGA
∆NS5-F ^a^	A9249G	I563V	GAT GGCCCCTCATCATAAAGTCCTAGC
∆NS5-R ^b^			GAAAATGGCTTTAGCTAGGACTTTATGATG

^a^ Forward primer; ^b^ reverse primer; ^c^ nucleotide substitution; ^d^ amino acid change. Underlined sequences represent the overlapping region and red letters are single-nucleotide substitutions used for site-directed mutagenesis by In-Fusion cloning.

**Table 2:** Sequence disparities between DENV-4 genomes in mosquito and human hosts.

Nucleotide *^a^*	Amino Acid *^b^*	Genome Region/Gene	Substitution Frequency (%)	
			Mosquito (*n* = 2)	Human (*n* = 11)
G186A	V29M	C	100	45.5
A657G	T73A	prM	100	90.9
G1602A	A222T	E	100	45.5
C1637T	Y233H	E	100	45.5
T2223C	F429L	E	100	90.9
T2437C	V5A	NS1	100	81.8
C2717A	T98S	NS1	100	90.9
T2746C	V108A	NS1	50	0
G2805A	A128T	NS1	100	90.9
C2880T	L153F	NS1	100	90.9
T3159G	S246A	NS1	100	81.8
A3721G *	K81R	NS2A	100	18.2
T3980G	I167M	NS2A	50	0
C4555T	A11V	NS3	50	0
A4574G	A17T	NS3	50	0
A4643G	I40V	NS3	50	0
A4843G *	K107R	NS3	100	0
G5484A	A321T	NS3	100	81.8
A5828T	P435T	NS3	100	81.8
A5962G	K480R	NS3	100	81.8
C8368	T269I	NS5	100	90.9
A9249G *	I563V	NS5	100	45.5
T9387A	L609M	NS5	100	63.6
G9467T	Q635H	NS5	100	90.9

*^a^* The position of nucleotide change in the genome (GenBank accession number NC_002640). *^b^* The position of amino acid substitution in the protein. * Specific mutation selected for *in vitro* viral gene functional study.

## Data Availability

All generated viral genome sequences acquired in this study were deposited into the NCBI sequence read archive (BioProject accession number PRJNA855615).

## Supplementary Materials

The following supporting information can be downloaded at: https://www.mdpi.com/article/10.3390/v15040982/s1, Figure S1: Selection pressures on the individual gene of DENV genome; Figure S2: Sanger sequencing results of the mutant viruses; Table S1: The primers for amplification of the whole DENV genome.
